# Neo-Hippocratic healthcare policies: professional or industrial healthcare delivery? A choice for doctors, patients, and their organisations

**DOI:** 10.1186/s12913-020-05890-3

**Published:** 2020-12-09

**Authors:** Jean-Pierre Unger, Ingrid Morales, Pierre De Paepe, Michel Roland

**Affiliations:** 1 Department of Public Health, Institute of Tropical Medicine, Nationalestraat 155, B-2000 Antwerp, Belgium; 2 Office de la Naissance et de l’Enfance, French Community of Belgium, Chaussée de Charleroi 95, B-1060 Brussels, Belgium; 3 Département de Médecine Générale, Université Libre de Bruxelles, Route de Lennik, 808, BP 612/1, B-1070 Brussels, Belgium

**Keywords:** Public services, Quality of health care, Healthcare accessibility, Non-profit health organisations, Medical professionalism, Health policy, Health systems

## Abstract

**Background:**

Ethical medical practice requires managing health services to promote professionalism and secure accessibility to care. Commercially financed and industrially managed services strain the physicians’ clinical autonomy and ethics because the industry’s profitability depends on commercial, clinical standardisation. Private insurance companies also reduce access to care whilst fragmenting and segmenting health systems. Against this background, given the powerful, symbolic significance of their common voice, physicians’ and patients’ organisations could effectively leverage together political parties and employers’ organisations to promote policies favouring access to professional care.

**Main text:**

To provide a foundation for negotiations between physicians’ and patients’ organisations, we propose policy principles derived from an analysis of rights-holders and duty-bearers’ stakes, i.e., patients, physicians and health professionals, and taxpayers. Their concerns are scrutinised from the standpoints of public health and right to health. Illustrated with post-WWII European policies, these principles are formulated as inputs for tentative action-research. The paper also identifies potential stumbling blocks for collective doctor/patient negotiations based on the authors’ personal experience.

The patients’ concerns are care accessibility, quality, and price. Those of physicians and other professionals are problem-solving capacity, autonomy, intellectual progress, ethics, work environment, and revenue. The majority of taxpayers have an interest in taxes being progressive and public spending on health regressive. Mutual aid associations tend to under-estimate the physician’s role in delivering care. Physicians’ organisations often disregard the mission of financing care and its impact on healthcare quality.

**Conclusion:**

The proposed physicians-patients’ alliance could promote policies in tune with professional ethics, prevent European policies’ putting industrial concerns above suffering and death, bar care financing from the ambit of international trade treaties, and foster international cooperation policies consistent with the principles that inspire the design of healthcare policies at home and so reduce international migration. To be credible partners in this alliance, physicians’ associations should promote a public health culture amongst their members and a team culture in healthcare services. To promote a universal health system, patients’ organisations should strive to represent universal health interests rather than those of patients with specific diseases, ethnic groups, or social classes.

## Background

The ethics embodied in the Hippocratic Oath was meant to govern the doctor-patient bond. Today, to achieve health, social, and professional goals, medical ethics needs to tackle tensions amongst patients, professionals, and the state.

We propose the term ‘neo-Hippocratic’ to define policies favourable to ethical medical practice and the universal right to professionally delivered health care. We propose to name (neo-) Hippocratic medical ethics those that are based on the values of “non-maleficence, beneficence, autonomy, and justice … the reference tetrad *par excellence* that physicians and ethicists use to resolve ethical dilemmas “ [1]. The ‘neo-‘prefix is justified by the distributive dimension that the quest for justice in making clinical and public health decisions adds to the traditional Hippocratic ethics. We leave it to the Ayurvedic or Chinese traditional practitioners to name their own proposed policies.

Medical values are guides for clinical and public health action. Value-based medical practice, say Pellegrino and Thomasma [1], is conditioned by the physician’s virtues. Self-effacement or altruistic benevolence is a virtue that expresses a predisposition to make beneficent clinical decisions. Compassion or empathy is the capacity to suffer with the patient. Compassion without self-effacement leads to the commercially-motivated medicalisation of psychosocial complaints.

Ethical medical practice requires professionally- and socially-minded health services and systems. When they are commercially financed and industrially managed, they strain the physicians’ autonomy and ethics because the industry’s profitability depends on commercial, clinical standardisation. Commercial insurance companies also reduce access to care whilst fragmenting and segmenting health systems [2].

Doctors’ associations (possibly, other health professionals’ organisations), mutual aid societies (social health insurance companies), and other organisations of non-specific patients are currently unimportant market forces. However, they could exert considerable political leverage by standing together and promoting, under the scrutiny of the news media, access to professional health care in universal health systems, for the following reasons:

- Their united voices could achieve considerable symbolic strength;
- everyone will be someday a patient and demand good care; and
- 15–20% of the population in high-income countries (HICs) works in the health sector.

In 2000, D.J. Rothman advocated alliances between professional associations and consumer groups “to accomplish goals that neither can realise separately” [3]. That same year, in pointing out threats to physicians’ self-regulation capacity, W.M. Sullivan [4] advocated putting morality at the core of efforts to re-inspire the contract between professionals and society and thus avoid inequality due to disease.

Still, social trust in physicians’ commitment to the public has continued to erode [5]. Despite the many pleas to protect the social contract enfranchising physicians, U.S. doctors have not resisted the commoditisation of care effectively: “The goal of maintaining technical expertise among physicians” [4] may have been exceptionally well met with regard to medical self-regulation. At the same time, healthcare professionals’ organisations may have overlooked political coordination with patients’ organisations.

What are the material and historical foundations of tentative doctor-patient alliances to amend healthcare policies in Europe?

Historically, European patients’ organisations have

- defended the (potentially contradictory) interests of general patients and of patients with specific health conditions;
- interfered in the role taken by the pharmaceutical industry in policy design;
- co-managed public finances (the Belgian health budget, for example, is co-managed by the major players in the sector, under government supervision); and
- in some rare cases, in line with primary healthcare philosophy, they co-managed public services, similar to what has been done in many low- and middle-income countries’ (LMICs’) government health centres.

Not all mutual aid societies and public services have fostered solidarity in health. Some have served specific, privileged socio-economic and/or ethnic groups and patients with special pathologies. Others have been accused of being bureaucratic and costly. In Belgium, however, the administrative costs of mutual aid societies are below 3.3% of their budgets, compared with often more than 20% in commercial insurance banks [6].

Meanwhile, physicians’ associations have

- secured self-regulation, a condition for the profession’s social enfranchisement;
- promoted doctors’ autonomy, status, and professional ethics;
- defended their members’ material interests; and
- organised continuing medical education

In several instances, professional organisations may have combined self-regulation and self-interest [7, 8], in opposition to professional ethics.

Whilst some mutual surveillance between patients’ (and in particular mutual aid funds) and doctors’ organisations was thus desirable, their collective relations in Europe were historically plagued by mutual distrust. Budgeting national health funds was the conflict-ridden topic that strained the dialogue about shared interests in health care policy.

To overcome this legacy and make an alliance possible, policy principles should be agreed between professionals’ organisations and patients’ mutual aid funds and other patients’ organisations. Those proposed here are formulated as proposals for action-research and thus fated to be amended by negotiations between them.

## Main text

To provide a foundation for negotiations between physicians’ and patients’ organisations, we propose health policy principles formulated as inputs for action-research. They are derived from an analysis of rights-holders and duty-bearers’ stakes, i.e., patients, physicians and health professionals, taxpayers, and the state. Their concerns and health stakes are scrutinised from the standpoints of public health and right-to-health concepts regarding quality and accessibility of individual, family and community care; physicians’ requirements to practice medicine professionally; and taxpayers’ interest in social justice in the healthcare sector. These principles are illustrated by post-WWII European policies. Based on the authors’ personal experience, the paper also identifies potential stumbling blocks for the proposed collective doctor/patient negotiations based on an analysis of the divergence of physicians’, patients’, and taxpayers’ concerns. A review of the literature in Pubmed with the terms *doctor*, *patient*, and *negotiation* did not yield a single article dealing with their collective bargaining (but 49 touching on person-to-person issues in clinical negotiation or issues specific to certain specialisations and pathologies).

## Result of analysis of rights-holders and duty-bearers’ stakes

### Patients’ stakes

#### Patients need medical practice to abide by defined care quality criteria

Patients’ organisations are entitled to having the quality of care used as the yardstick for government health care policies, and they actually often do make that demand. How in that case may quality of care be defined? We propose the following six categories:

- *eco-biopsychosocial*. Since G.L. Engel’s seminal work [9], doctors are expected to address the patient’s suffering, health risks, and objectives whilst linking biomedical decisions to the patient’s psychology and social determinants. They should do this with empathy and self-effacement. However, whilst the control of social and biological determinants in medical practice benefits from a degree of standardisation [10, 11], the psychological care component does not appear to be very compatible with standardisation [12]. Even the simple standardisation of the measurement of the quality of mental health care raises problems that are difficult to overcome. Key barriers to this effort include lack of standardised information technology-based data sources and limited scientific evidence for mental health quality measures [13]. Although embodied AI [14] is said to address high-level therapeutic interventions that used to be offered exclusively by highly trained, skilled health professionals such as psychotherapists [15], the reality is less palatable: it consists today of AI-enabled, empathetic, and evidence-driven conversational mobile app technologies that should never replace time with a health care professional for more severe mental health problems [16]. The reason is that to be eco-biopsychosocial, health care should be largely bespoke. We use the term “industrialised care” to refer to those types of care that cannot be adjusted to individual patients and family needs and features. Commercial financing leads to the industrialisation of care because standardisation allows economies of scale and orients medical and pharmaceutical consumption.
- *Person-centred*. In the U.S., the Institute of Medicine has defined this concept as, “Providing care that is respectful of and responsive to individual patient health goals, needs, and values, and ensuring that her/his values guide all clinical decisions.” [17]
  Along the same lines, Angela Coulter [18] defines patient engagement as the relationship between patients and care providers to “promote and support active patient and public involvement in health and healthcare and to strengthen their influence on healthcare decisions, at both the individual and collective levels.”
  In practice, person-centred care delivery assumes doctor-patient negotiation of clinical conduct. Care delivery cannot truly be eco-biopsychosocial if the doctor’s advice on changing the patient’s way of life is not negotiated with her/him.
- *Skilled*. Decent healthcare delivery requires manual, behavioural, and communication skills (for instance, to break bad news to a patient). They cannot be learned exclusively from books. That is why oral tradition, demonstrations, and role models have been so important in transmitting medical professionalism ever since the ancient Greek and Gaelic cultures.
- *Perfectible*. Physicians should systematically aim to identify and correct their mistakes (which are inherent in human activities) with reflective methods, continuing medical education, and teamwork, so that they become the starting point of a learning process.
- *Scientific*. Biology-based clinical decision-making also requires sufficient autonomy in interpreting clinical standard operating procedures (SOPs) because “the evidence-based quality mark has been misappropriated by vested interests, the volume of evidence has become unmanageable and EBM guidelines often map poorly onto complex multi-morbidity” [19]. Clinical trials, systematic reviews, and meta-analyses should not obscure the other components of EBM, namely, the doctor’s experience and the patient’s context [20]. Another consequence is that physicians should learn to read scientific papers and clinical guidelines critically.
- *Ethical*. Physicians (and other health professionals) must adhere to a code of professional ethics and this code must be updated regularly through jurisprudential discussions in health services and professional associations and dialogue with other professionals or patients.

In sum, with regard to the quality of care, patients’ organisations are justified in demanding access to care that is delivered professionally in universal health systems and policies to disseminate professionalism in health services. Health policies should be professionally minded if they are to be socially driven.

#### Patients need professional, not industrial, health care

Patients’ and taxpayers’ organisations are entitled to demand that physicians optimise their impact on collective health. However, such optimisation paradoxically requires highly individualised care, since physicians must address individual, family, and community eco-biopsychosocial health risks that are prioritised with the patient. This puts a conceptual limit on the standardisation of health care. Although useful, no computer programme, however sophisticated, will replace doctors in the near future, and this will hold true as long as ethics permeates medical practice. Mere industrial products per se will not secure the universal right to care.

Merriam Webster defines managed care as follows: “a system of providing healthcare (as by a Health Maintenance Organization or a Preferred Provider Organization) that is designed to control costs through managed programs in which the physician accepts constraints on the amount charged for medical care and the patient is limited in the choice of a physician”. Managed care began spreading across the U.S. more than thirty years ago [21]. The corollary was the loss of a “guild monopoly” where it was implemented [4].

Arguably, commercially managed care strains medical professionalism because of the following:

- It misuses EBM subject to a restrictive interpretation that overlooks the patient’s eco-biopsychosocial features, her/his life goals, the doctors’ values, and the idiosyncrasies of doctor-patient communication. Health insurance companies elicit and often impose a literal, normative use of clinical guidelines and so reduce the professional’s scope for interpretation and doctor-patient shared decision-making on clinical conduct.
- In prompting suggestions to improve evidence production and interpretation [22], clinical guidelines reflect *for-profit* trade-offs between opposing values (such as cost control v. patient well-being, the quest for the patient’s autonomy v. security, and efficiency v. effectiveness), thus to the detriment of the patient [23].
- Managed care undermines the eco-biopsychosocial approach and the doctor’s self-effacement, especially if it relies on fee-for-service and pay-for-performance schemes.
- Commercial insurance companies make access to (high-tech) care contingent on the patient’s purchasing power, thereby maximising the consumption of care by the wealthy and minimising the consumption of care by the poor or vulnerable groups.

On an ethical level, the industry undermines the quality of care through managerial efforts to shift the physician’s motivation from intangible to material and from qualitative to quantitative incentives (for instance with Pay-for-Performance remuneration), albeit without managing to achieve an impact on health care outcomes [24]. Furthermore, since actuarial management places tight limits on risk pooling to regulate access to expensive technologies, it needs unlimited access to patient information (genetic information, for instance) to assess patient risks. Commercial medical practice thus comes into conflict with this other Hippocratic tenet: “Whatsoever I shall see or hear in the course of my profession, as well as outside my profession in my intercourse with men, if it be what should not be published abroad, I will never divulge, holding such things to be holy secrets”.

To overcome the obstacle of professional ethics, managed care echoes the physician’s technical identity in order to encourage her/his reliance on medical technology and pharmaceuticals. This marketing strategy weakens the doctor’s professional identity by undermining the credibility of any knowledge not validated by quantitative, probabilistic science, e.g., knowledge useful for ethical, eco-biopsychosocial, and person-centred medical practice.

Whilst commercial healthcare financing erodes the ethical foundation of medical practice irremediably, public services are not immune to excessive standardisation of care and unethical practice:

- In Europe, laws sometimes permitted commercial deals between physicians and labs or pharmacies, and such deals were even struck in government services [25].
- Medical technology and pharmaceutical industries often paid public services’ physicians to prescribe inefficient products.
- Practitioners multiplied unneeded procedures, admissions, and consultations to take advantage of national health insurance “diagnosis related groups” (DRGs).
- Physicians with dual public/private practices may have self-referred profitable patients.

Therefore, public services must be regulated, controlled, and financed to promote professional ethics. Public health programmes that operate through the provision of clinical care should promote its bespoke, customised delivery.

### Communities need access to clinical medicine but also community medicine and public health programmes

Communities need sufficient access to family medicine (or its equivalents) and to hospitals, both specialised and general, peripheral and academic. For this, health policies must remove all kinds of obstacle: financial (medical debts are one of the biggest sources of debt in the United States), geographical, temporal, cultural, psychological, technical, administrative, pharmaceutical, etc.

Communities actually benefit from physicians’ attempts to maximise their collective impact whilst individualising the delivery of care [26]. Besides this, persons with health risks need to be able to access

- disease-, syndrome- or risk-specific public health programmes and
- community-oriented primary care.
  The latter is important to be able to act upon the social determinants of health that are susceptible to community and possibly intersectorial interventions. For instance, in the US women from minority neighborhoods have the smallest babies and the largest problems with care. They are especially in need of public health programs, and it is precisely the disadvantaged who tend to have the least access to them. The following are examples of its achievements: grain storehouses to reduce infant malnutrition in West Africa, psychosocial support for AIDS orphans in Central Africa, shelters for battered women in Europe, and school drop-out prevention for teenagers in Latin America.

The commoditisation of care strains the integration of medicine and public health because commercial insurance banks and industrial healthcare services see only opportunity costs in the public health externalities that clinical practice can achieve. This is why they discard

- disease-control interventions, because they often lack profitability [27].
- integrated prevention, because it requires lengthier consultations to analyse the patient’s environment. Notice that task-shifting may sometimes provide a solution in the context of teamwork (https://ec.europa.eu/health/expert_panel/sites/expertpanel/files/023_taskshifting_en.pdf).
- professional development and physicians’ involvement in organising health services, because non-clinical medical activities are viewed as long-term investments generating immediate opportunity costs.

### Physicians’ stakes

Since WWII, most European physicians have had meaningful, rewarding professional lives and decent incomes. However, ahead of legal changes to commoditise health care, physicians were put under workload and financial stress in public hospitals whilst being offered tempting commercial revenue from private hospitals and insurance companies.

#### Physicians need meaning, recognition, sharing, and dignity

Professional proficiency is a source of social recognition. Teamwork makes it possible to share knowledge and experience. Ethical medical practice is a source of pride and intangible (non material, or symbolic) motivation. However, there are reasons to believe that under the rule of commercial health insurance schemes physicians feel a loss of values, something that Durkheim called “anomia”. First, private insurance companies bureaucratise medical practice. The U.S. health industry boasted 1.5 million white-collar workers busy limiting access to care and imposing clinical conduct and extensive clinical data collection on barely half as many doctors [28] because actuarial management needs a wealth of details to cost insurance and price care. Second, they undermine the physician’s intangible motivation to practice ethically. A Medscape Report [29] disclosed that 32% of American physicians regularly had to discuss costs with their patients, adding to the burden imposed by the growth of paperwork in medical practice. Burnout in physicians is caused not only by financial and workload issues, but by industrial care policies that are conducive to unethical medical practice [30], de-professionalisation, and professional acculturation.

It is tempting to posit that intangible benefits such as autonomy and recognition carry little weight compared with material incentives. That would be a mistake. American doctors are amongst the highest paid in the world. However, in the U.S., the suicide rate of physicians is the highest of all professions, greater than the military suicide rate and almost twice the national average [31]. According to the same 2014 report, 42% of U.S. physicians would not have chosen medicine as a career had they been given the opportunity to start over [32], whereas 2 years later 54.4% of U.S. physicians were reporting that they felt burnt out [33] because of high workloads and disappointment with their professional practice. This burnout figure in a fully mature market contrasts with 30.2% in Madrid in 2003 [34] and 16% in Switzerland in 2005 [35], although the differences must be interpreted with caution. It is even more significant that today the burnout rate of European doctors has become comparable to what it is in the U.S. [36].

#### Physicians need decent incomes and workloads

Commercial insurance companies often promise doctors material benefits. European doctors need to learn that in spite of the apparently high compensation in some U.S. specialities, Western European doctors are comparatively well paid.

First, with female doctors earning about 25% less than their male colleagues, only 50% of U.S. physicians felt fairly compensated in 2014 [29, 37].

Then, consider a 2014 international comparison of doctors’ compensations in health market and non-market countries [38]. Admittedly, three of the four largest health insurance markets in HICs (USA, Australia, and the Netherlands) also gave their specialists the highest average yearly compensations both in absolute terms (230,000, 247,000, and 253,000 U.S. dollars, respectively) and as a percentage of per capita GDP (5.6, 7.6, and 6%, respectively). This, however, is no reason to view health markets as being synonymous with the highest compensation for specialists, for in 2014, Swiss specialists were earning US$130,000, or less than specialists in Ireland (US$143,000), France (US$149,000), the UK (US$150,000), and Belgium (US$188,000). What is more, income inequality between specialist categories was probably the highest in the U.S., with orthopaedists earning US$413,000 a year against US$188,000 for internal medicine physicians and US$181,000 for paediatricians [39]. At the same time, Dutch (with US$117000) and Swiss (with US$116,000) GPs were earning no more than their British counterparts ($118,000). All in all, the specialist/GP compensation ratio was less favourable to GPs in Australia (0.4), the Netherlands (0.6), and the U.S. (0.7) than in Germany and the UK (0.8).

Notice that several parameters artificially smooth out the differences in physicians’ compensations in health market v. non-market countries. Money conversion uncertainties lead to inaccuracies. Several factors reduce the difference between physicians’ incomes in market v. non-market countries: the failure to take account of huge malpractice insurance costs (up to US$120,000 for some specialities in the U.S.); medical education costs; physicians’ household expenditures on health; and children’s education, which is much more expensive in the U.S. than in the EU (where public services, including education, are better and cheaper).

### Taxpayers’ stakes

Taxpayers need equitable, efficient health systems at home and international cooperation in health that favours equitable health systems abroad. Alongside direct payments, patients also contribute to health systems through their income taxes. Given that efficiency is a condition of equity and social justice, they should worry that their taxes are not used efficiently by commercial health systems [40].

From the moment health insurance was privatised in the Netherlands (2006) and Switzerland (1996), these two countries joined the U.S. in having the highest government health expenditures in the OECD. This trend held until 2013, after which there has been conflicting evidence as to a possible stabilisation in the Netherlands. U.S. per capita public health expenditure alone was higher than total health expenditure in most EU countries, whilst rich Americans had more difficulty accessing care than the poor in many European countries [41]. The inefficiency of the U.S. system was such that between 1999 and 2009, “Although family income grew throughout the decade, the financial benefits that the [U.S.] family might have realised were largely consumed by healthcare cost growth, leaving them with only $95 more per month than in 1999.” [42].

The U.S., where the healthcare market has achieved full maturity, has the largest rich/poor life expectancy gap in the industrial world, shortening life expectancy for the third consecutive year, increasing maternal mortality for the past twenty years, and very limited or no access to healthcare for 10% of its population [43].

HIC (high-income country) taxpayers would be well advised to demand the re-engineering of international cooperation in health, if not for the human right to care, out of self-interest: to stabilise LMICs politically and militarily, to control epidemics, and to reduce migration pressure. International cooperation should promote access to professional care in universal health systems, as in Europe, and not limit health cooperation to disease control on the grounds of its alleged efficiency [44]. Bilateral cooperation agencies should rely to a much greater extent on the medical expertise available in Europe. If, as WHO claims, the international community is serious about strengthening health systems in LMICs and Europe is serious about reducing migration pressure, government agencies should stop contracting out cooperation activities to agents with possible conflicts of interests, such as foundations created and funded by industries. In the U.S. at least, these industries are legally obligated to make a profit and open up health markets to competition [45], possibly to the detriment of access to care.

As a conclusion of this analysis, the majority of doctors and patients would benefit from a policy of universal access to professional care. However, the efficiency and social equity of the health system is a concern of patients and taxpayers, not necessarily of physicians. To get physicians’ political support for publicly-oriented health systems, public financing must ensure decent incomes for professionals in socially-minded medical practice and public policies must appeal to the physician’s intangible motivation.

### Principles of neo-Hippocratic health care policies: driven by concern for social welfare and professionalism

To formulate policy principles consistent with the right to care and physicians’ intangible and material motivations, we shall now try to reconcile doctors’ and patients’ concerns whilst relying on public health standards. These policies address the human right to health care; medical professionalism and ethics; and social justice in health policy.

a Policies for the human right to health care: the right to access professionally-delivered care in universal health systems

Universal health systems encompass the entire array of services, from family medicine to university teaching hospitals, acute to chronic care units, and polyclinics to highly specialised hospitals.

1. Patients’ organisations would be well advised to demand that access to professionally- delivered care in universal health systems should be treated as a human right because denying it amounts, in public health terms, to inflicting avoidable suffering, anxiety, and mortality risks. In addition, care has been shown to be an important health determinant – perhaps the most important *single* determinant – in high-income countries (HICs) [46] and LMICs alike [47].

Patients and persons with health risks are entitled to demand easy access to

- general practitioners/family physician (they are used interchangeably in this paper) because they are close to the patients’ homes; they can provide eco- biopsychosocial care and individually tailored prevention; and improve the patient’s environment (by reducing domestic violence, for instance) [48, 49]. They can solve more than 90% of new health problems presenting in first-line health services. And they can do this efficiently, since for a same outcome, the cost of treating, say, common diarrhoea increases with the complexity of the accessed health infrastructure.
- hospitals and consultants, in order to access specialised expertise, technology, surveillance, and emergency support. In 1998, GP contacts represented 70–90% of all patient contacts with England’s NHS [50]. Some twenty years later – in 2017–2018 – England, with an ageing population, logged 16.6 million finished admission episodes and 20 million contacts with consultants for a population of 55.8 million.

From a public health perspective, all patients should access the same range of hospitals because the risks of avoidable mortality, suffering, and anxiety are what ought to define service utilisation. Consider maternal mortality. In LMICs, the maternal mortality rate is known to depend on access to skilled birth attendants, first-line maternities, and emergency obstetric services [51]. Lowering the MMR further below standards in most LMICs also requires access to regional and university teaching hospitals to treat complicated cases of embolism, gestational diabetes, and multi-resistant puerperal sepsis and to perform early caesarean sections.

Whether out of self-interest or on other grounds, physicians’ organisations ought to support policies that tackle the multiple dimensions of care accessibility and timeliness. Instead, the WHO Universal Health Coverage (UHC) strategy addresses only the financial dimension of care accessibility, not the geographical, administrative, social, technical, psychological, and cultural obstacles to care, and thus possibly to the detriment of removing such obstacles. Besides this, for the sake of public health, governments should monitor population-based service utilisation indices and disease-specific early detection and continuity rates.

Patients, many physicians’ organisations, and taxpayers will probably hold similar views on other policy characteristics.

2. Hospitals should strengthen the entire health system with evaluations of first-line services, continuing medical education, action- and operational research, and sharing their medical equipment.
3 Policies should promote public health activities in clinical practice and, symmetrically, individual care delivery in community and public health medicine.
4 Services should be managed and planned as if they formed a system. To be managed *systemically*, health services should have the following features: complementarity of their tiers’ functions; absence of functional deficiencies in the set of services offered; access to the needed tier; patient follow up by clinical information (for instance, integrated electronic records with a compatible classification system) [52]; GPs as gatekeepers, in charge of the patient’s eco-biopsychosocial synthesis and providing advice on health service utilisation; and the optimisation of medical technique allocation over the health service continuum.
5 Health care services should have a public interest mission, to wit: they should be unique, mandatory, based on solidarity, and prohibiting segmentation (thus not specific to social classes) and exclusion (for example, on the basis of risk selection). Notice that this Belgian law defining publicly-oriented health financing is a practical model for Europe because the European Court of Justice has validated it.
6 Doctors’ and patients’ organisations will probably find common cause in opposing austerity policies applied to health care and international trade agreements, such as CETA and TISA, that address investments in health care, because such agreements favour the insurance banks’ control over healthcare management [53]. International private arbitration courts should not be allowed to replace the European Court of Justice because such a move would bury the European jurisprudence that immunises publicly-oriented services and mutual aid societies from free competition and investment laws.

#### Policy principles for medical professionalism and ethics

The following principles are derived from public health considerations. Their “doctor-patient negotiation” may be complicated but not insurmountable.

7. Health management should be not-for-profit.
8 Policies should favour professional practice and make it consistent with a code of ethics that is updated in particular to incorporate medical and public health concerns. In Belgium, for instance, the government finances periodic medical ethics seminars in health services.
9 To steer doctors’ intangible motivations and transmit professional knowledge, medical faculties and health services should transmit a culture with values, different viewpoints, and their validation, not mere competences as envisaged by managerial ideologies (https://ec.europa.eu/health/expert_panel/sites/expertpanel/files/docsdir/024_defining-value-vbhc_en.pdf), [54]. To achieve this, an array of non-clinical medical activities is available: stabilisation of doctor- patient relationships through a territorial definition or a list system; technical/psychological doctor coaching by experienced professionals (in Spain, experienced specialists have long offered younger colleagues technical and psychological support); internal (and external) audits [55]; continuing medical education; clinical coordination; inter-professional teamwork [56]; and adhocratic organisation [57]. Health policies should support non-clinical medical activities and the doctor’s ‘manager-physician’ role, which connects all these activities, in particular.
10 Pre-graduate and continuous medical education should also transmit clinical skills. A decade ago, medicine professors were still selected in Scandinavian countries on the basis of not just their publications, but their clinical aptitudes as well.
11 Health policies should ensure decent incomes for physicians and health professionals but refrain from tying remuneration to clinical decision-making (as per pay for performance (PFP) schemes) in order to avoid focusing MDs’ attention on income when they make clinical decisions [58]. Indeed, PFP has been proven to reduce care quality for apparent efficiency gains [59]. Thanks to various mixes of capitation, salaries, and fee-for-service schemes, European physicians have long escaped overly material incentives.
12 Physicians who play key roles in hospital management give rise to the best hospital performances [60]. They should have a pivotal role in service and system organisation in order to link care management to clinical decision-making.
13 To give doctors a sense of “health system ownership”, physicians should be permitted to co-manage the health system. For instance, since the late 1990s, volunteer physicians in Denmark have participated in designing national healthcare guidelines in dialogue with Odense University [61].

#### Social justice in health policy

14. Healthcare policies should abide by the principle of solidarity and mobilise sufficient contributions from the rich to universal health systems so as to enable the poor and the middle class to access professional care [62]. Solidarity in health assumes sufficient total spending on health (in 2016, Belgium and the Netherlands were spending about 10% of their GDPs on health) together with sufficient public expenditure on health. History tells us that 80% of total spending on health sustained good access to universal health systems in Europe for decades. Room for manoeuvre still exist. In 2016, the expenditures of government/compulsory schemes amounted to 8.4% of GDP in the Netherlands against only 7.9% in Belgium [63].
  Policies can rely on either publicly-oriented single-payer Bismarkian schemes (as in France or Belgium before 2000) or Beveridgean government healthcare services (as in the UK, Italy, Spain, and Sweden before 2000) to secure non-profit health management. In any case, however, the health insurance market should be severely constrained by a sufficient *proportion* of public expenditure on health and strong regulations. International trade treaties should not be permitted to address health care financing.

Action-research is needed to test the validity and relevance of the analysis and principles discussed here in various contexts.

Applying public health criteria to assess healthcare policies is politically effective because they enable political parties to evoke the large numbers of death and suffering that proper healthcare policies can avoid. Using these criteria in health systems research reveals that commercial health financing hampers patients’ access to professional health care, strains patients’ relationships of trust with their doctors, and reduces doctors’ autonomy.

## Conclusion

Doctors’ associations need to engage in a policy dialogue with patients’ organisations committed to the universal right to care in order to formulate policies barring commercial health insurance companies from competing for public funds with professionals, patients, and publicly-oriented services. The principles presented here represent a tentative platform for such negotiations. Whilst patients’ and physicians’ organisations in Europe have many common concerns, they may also collide on some issues:

- Mutual aid associations need to recognise the pivotal role of physicians in multidisciplinary teams, as physicians generally are the only professionals capable of assessing the patient’s eco-biopsychosocial status and help multidisciplinary teams to address their needs.
- Physicians’ organisations need to recognise the not-for-profit mission of healthcare financing and management as a condition of ethical, professional practice, and the importance of patients’ associations in policy design and service management.
- Doctors’ organisations must recognise the public health importance of access to health care for all social strata, ethnic groups, and isolated populations and act to improve such access both at the political level and in relations with their members.
- Mutual societies also need to recognise that ethical medical practice is contingent on providing doctors with sufficient professional autonomy and income.
- Doctors’ organisations should explain to patients‘ associations how they manage the ethical risks associated with their autonomy and how, beyond enforcing professional regulations, they aim to motivate, educate, and assess practitioners.

Physicians’ professional organisations will also have to promote a public health culture amongst their member clinicians and a clinical culture in public health and publicly-oriented health services.

In promoting neo-Hippocratic policies, patients’ and health professionals’ organisations may find unexpected allies. In a context of long-term economic stagnation, entrepreneurs who do not invest in health (say, 80% of all investors) would do well to check health expenditure trends in the U.S., the Netherlands, and Switzerland twice rather than once. This would help them to assess their likely losses in the coming decade if healthcare financing is commoditised in Europe.

The existential threat that the industrialisation of care poses to medical professionalism could favour patients’ and doctors’ organisations’ agreeing on national policies propitious for accessing professional care in universal health systems.

## Data Availability

Data sharing is not applicable to this article as no datasets were generated or analysed during the current study.

## Abbreviations

CETA: EU-Canada Comprehensive Economic and Trade Agreement
DRG: Diagnosis Related Groups
EBM: Evidence-Based Medicine
GP: General Practitioner
HIC: High Income Countries
HMO: Health Maintenance Organization
LMIC: Low and Middle Income Countries
MMR: Maternal Mortality Rate
PFP: Pay-for-Performance
TISA: Trade in Services Agreement
UHC: Universal Health Coverage
